# Supplementary material for: Identification and functional analysis of non-coding regulatory small RNA FenSr3 in Bacillus amyloliquefaciens LPB-18
Source: PeerJ. 2023 May 15;11:e15236. doi: 10.7717/peerj.15236 (PMC10194069; doi:10.7717/peerj.15236)
Supplement: Supplemental Information 4 [file peerj-11-15236-s004.zip › KO/CK-vs-T1_map/map00680.html]

KEGG PATHWAY: Methane metabolism - Reference pathway


|  |  |
| --- | --- |
| **Methane metabolism - Reference pathway** |  |

[
Pathway menu
| Organism menu
| Pathway entry
| Show description
| User data mapping
]

Methane is metabolized principally by methanotrophs and methanogens in the global carbon cycle. Methanotrophs consume methane as the only source of carbon, while methanogens produce methane as a metabolic byproduct. Methylotrophs, which are microorganisms that can obtain energy for growth by oxidizing one-carbon compounds, such as methanol and methane, are situated between methanotrophs and methanogens. Methanogens can obtain energy for growth by converting a limited number of substrates to methane under anaerobic conditions. Three types of methanogenic pathways are known: CO2 to methane [MD:M00567], methanol to methane [MD:M00356], and acetate to methane [MD:M00357]. Methanogens use 2-mercaptoethanesulfonate (CoM; coenzyme M) as the terminal methyl carrier in methanogenesis and have four enzymes for CoM biosynthesis [MD:M00358]. Coenzyme B-Coenzyme M heterodisulfide reductase (Hdr), requiring for the final reaction steps of methanogenic pathway, is divided into two types: cytoplasmic HdrABC in most methanogens and membrane-bound HdrED in Methanosarcina species. In methanotrophs and methyltrophs methane is oxidized to form formaldehyde, which is at the diverging point for further oxidation to CO2 for energy source and assimilation for biosynthesis. There are three pathways that convert formaldehyde to C2 or C3 compounds: serine pathway [MD:M00346], ribulose monophosphate pathway [MD:M00345], and xylulose monophosphate pathway [MD:M00344]. The first two pathways are found in prokaryotes and the third is found in yeast. As a special case of methylotrophs, various amines can be used as carbon sources in trimethylamine metabolism [MD:M00563].

|  |  |  |
| --- | --- | --- |
| Reference pathway | 184% 150% 122% 100% 82% 67% 55% | 图片下载 |
